# Triple therapy after PCI – Warfarin treatment quality and bleeding risk

**DOI:** 10.1371/journal.pone.0209187

**Published:** 2018-12-18

**Authors:** Daniel Wadell, Jens Jensen, Erling Englund, Anders Själander

**Affiliations:** 1 Department of Public Health and Clinical Medicine, Umeå University, Sundsvall, Sweden; 2 Department of Medicine, S:t Görans hospital, Karolinska Institute, Stockholm, Sweden; 3 Department of Research and Development, Region Västernorrland, Sundsvall, Sweden; University of Massachusetts Medical School, UNITED STATES

## Abstract

**Background:**

A combination of warfarin, aspirin and clopidogrel is indicated after percutaneous coronary intervention (PCI) in some patients, despite the higher risk of bleeding inferred by this triple therapy.

**Objectives:**

Whether the treatment quality of warfarin measured by iTTR (individual time within therapeutic INR range) is associated with bleeding complications during triple therapy after PCI.

**Methods:**

A retrospective register study consisting of 601 triple treated PCI patients from the Swedish Coronary Angiography and Angioplasty Registry (SCAAR). The cohort was cross-matched with the Swedish Patient Registry for background characteristics and bleeding complications up to 6 months after PCI using ICD10 codes, the Prescribed Drug Registry for ongoing medications, and the national oral anticoagulation registry Auricula for warfarin treatment quality. The patients were grouped into four iTTR groups: <50%, 50–69.9%, 70–84.9% and >85% as well as iTTR above or below 70%.

**Results:**

Of 601 patients, 39 (6.5%) had a bleeding complication (type 2 according to BARC). Bleeding was more common for iTTR<70% compared to iTTR>70%, 28 (9.3%) vs. 11 (3.7%) (p = 0.005). The bleeding frequency increased gradually from the best group, iTTR>85% with four bleeders (3.3%) up to 17 bleeders (13.3%) in the worst group with iTTR<50% (p = 0.003), with a corresponding bleeding rate per 100 treatment years of 8.0 and 44.9, respectively. In multivariate analysis low BMI, HR 1.11 (95% CI 1.01–1.22), a medical history of anemia HR 3.17 (1.16–8.69) and iTTR < 70% HR 2.86 (1.25–6.53) increased the risk of bleeding.

**Conclusion:**

Triple therapy after PCI confers a high risk of bleeding events. Warfarin treatment quality measured by iTTR as well as a medical history of anemia are strong independent predictors of bleeding in these patients. Physicians should pay more attention to iTTR after PCI.

## Introduction

### Anticoagulation

The vitamin K antagonist, warfarin, is the only oral anticoagulant that is approved for all common indications to anticoagulation (atrial fibrillation, recent or repeated pulmonary embolism, cardiac embolism and mechanical heart valve prosthesis) and is the anticoagulant still most commonly used in combination with antiplatelet therapy after PCI.

### Triple therapy after PCI

To reduce the risk of stent thrombosis and thereby myocardial infarction and death, double platelet inhibition (aspirin and ADP receptor antagonists such as clopidogrel, prasugrel or ticagrelor) is the recommended treatment after PCI. Atrial fibrillation or some other indication for anticoagulation therapy is not uncommon among patients with coronary heart disease and thereby combinations of antithrombotic treatments occurs [1–3]. Previous studies have shown a significantly increased risk of bleeding if patients take three drugs simultaneously (warfarin, aspirin and clopidogrel), so-called "triple therapy" [3–6]. The current medication strategy after PCI for patients also on anticoagulation therapy is to balance between the risk of thromboembolic events and the risk of bleeding complications by limiting the time on triple therapy or omitting aspirin [7].

### Individual time in therapeutic range

"Individual Time in Therapeutic Range" (iTTR) is an established measurement of the warfarin treatment quality regarding a certain patient [8, 9]. ITTR describes the percentage of time within the patients intended International Normalized Ratio (INR) target range, most commonly INR 2.0–3.0. Sweden displays by international comparison a high warfarin treatment quality partly due to the use of a national computerised dosing system, Auricula [10–12]. Auricula has a web-based dosing algorithm with dosage suggestions that result in higher iTTR than manual dosing [13, 14].

Two studies have published results of how iTTR affect the risk of bleeding after PCI treatment. One of these showed that iTTR had no bearing on the risk of bleeding and the other study showed a numeric but not statistically significant effect [15, 16].

For patients with atrial fibrillation, venous thromboembolism or mechanical heart valve prostheses on warfarin, iTTR has been shown to be important regarding the risk of bleeding [17–21]. Our aim is to study if iTTR is also associated with the rate of bleeding complications in triple therapy after PCI.

## Methods

A retrospective register study in which bleeding complications are studied in patients undergoing PCI and who have concomitant treatment with warfarin.

### Ethics

The study complies with the Declaration of Helsinki. The outcome of the patients was studied through anonymous data in national registries. Participation did not involve any potential risks or complications for the patients included in the project.

The Regional Ethical Review Board in Umeå approved the study.

Reference number: 2013 / 244–31

### Included registries

Auricula is a Swedish national quality register for atrial fibrillation and oral anticoagulation[22]. It was instituted in 2006 and now includes over 126 000 patients and more than seven million INR samples. It is updated daily and more than two hundred centers in Sweden work with the system. Examples of data are: indications, treatment time, INR values and given doses and discontinuations.

SCAAR (Swedish Coronary Angiography and Angioplasty Registry) is part of the "Swedish Web System for Enhancement and Development of Evidence-Based Care in Heart Disease Evaluated According To Recommended Therapies" (SWEDEHEART)[23]. Each year approximately 20 000 PCI are performed in Sweden. All PCI since 1999 are registered in SCAAR. A very large amount of patient-related data can be retrieved from the registry. Monitoring and control of register data are performed annually in all hospitals and compliance with the patients' hospital records for the study period was 95.5% [24].

The Swedish National Patient Registry (NPR) has since 1987 registered diagnoses according to the International Classification of Disease, 10th edition (ICD-10) for patients who were in hospital clinics or discharged from hospital care [25]. The registry is considered suitable for epidemiological research by The National Board of Health and Welfare and has a high penetration with lack of main diagnosis in 0.5–0.9% of cases [25, 26].

The Prescribed Drug Registry contains information on all prescribed medications, date of prescription and purchases, dosage and quantity from 1999 onwards in Sweden. Missing data for the registry is 0,02–0.6%[27].

### Population

**Fig 1:**
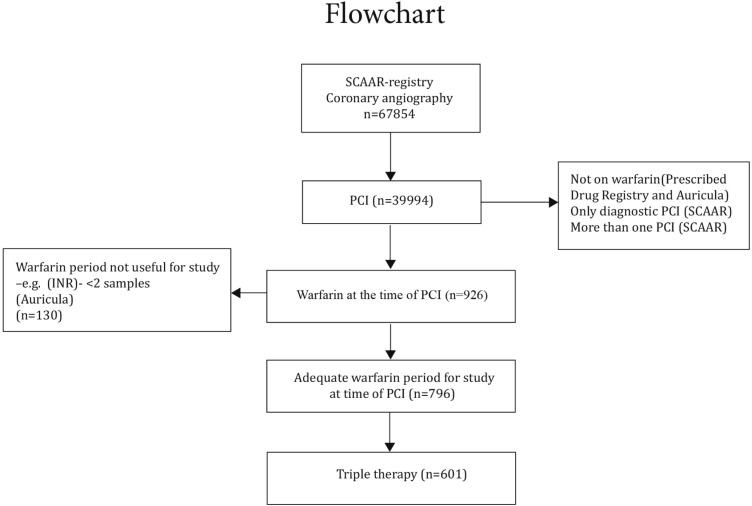
Trial profile.

The primary patient inclusion was made from the SCAAR registry and consisted of all PCI patients from 2010-01-01 to 2011-12-31 (n = 39 994). The inclusion and exclusion criterias are schematically presented in a flowchart (Fig 1). Patients without therapeutic PCI (i.e. only diagnostic PCI e.g. fractional flow reserve (FFR) or intravascular ultrasound (IVUS)) were excluded. If a patient had undergone more than one PCI, only the first intervention that also included a period with warfarin therapy in the Auricula registry was selected (n = 926). For inclusion, individuals also needed to have at least two INR values from the time of the intervention (PCI) up to six months (183 days) afterwards or until a bleeding complication occurred (n = 796). A six-months follow-up period after PCI was chosen because this was the most common period with combination therapy in Sweden at the time. A longer follow-up period would have increased the risk of including patients not on triple therapy to the study. To further reduce the risk of getting patients without triple therapy into the triple treated cohort we checked which individuals actually purchased warfarin, aspirin and clopidogrel from the pharmacy anytime between 100 days before up to 31 days after PCI in the Prescribed Drug Registry. The final cohort consisted of 601 patients with triple therapy (warfarin, aspirin and clopidogrel). In addition to the main cohort of triple therapy there were 100 patients with warfarin and clopidogrel, 55 with warfarin and aspirin and 40 patients with warfarin alone (Table 1). In the main cohort of triple therapy, only those with values on all variables in Table 2 were selected (432 cases), for the univariate and multivariate hazard ratio analysis.

**Table 1:** Background data for the original cohort based on antithrombotic therapy and iTTR.

Antithrombotic therapy	Total n = 796	Not on triple therapy* n = 195	Triple therapy n = 601	p	Triple therapy n = 601		p
					iTTR<70 n = 302	iTTR≥70 n = 299	
Background data							
Age at PCI	70.5 ±9.2	71.0 ±9.3	70.4 ±9.2	ns	69.5 ±9.9	71.3 ±8.4	0.02
Female gender	192 (24.1)	48 (24.6)	144 (24.0)	ns	78 (25.8)	66 (22.1)	ns
History of smoking	426 (53.5)	109 (55.9)	317 (52.7)	ns	160 (53.0)	157 (52.7)	ns
Creatinine clearance (ml/min)★	73 (57–93)	70 (55–86)	74 (57–94)	ns	73 (57–95)	74 (57–92)	ns
BMI	28.0 ±4.7	27.8 ±4.6	28.1 ±4.7	ns	28.0 ±4.6	28.2 ±4.7	ns
CHA2DS2-VASc	4 (3–5)	4 (3–5)	4 (3–5)	ns	4 (3–5)	4 (3–5)	ns
**Medical history**							
Heart failure	323 (40.6)	86 (44.1)	237 (39.4)	ns	126 (41.7)	111 (37.1)	ns
Hypertension	557 (70.5)	133 (68.2)	424 (70.5)	ns	210 (69.5)	214 (71.6)	ns
Diabetes	203 (25.5)	49 (25.1))	154 (25.6)	ns	79 (26.2)	75 (25.1)	ns
Stroke/TIA	171 (21.5)	33 (16.9)	138 (23.0)	ns	65 (21.5)	73 (24.4)	ns
Vascular disease#	609 (76.5)	150 (76.9)	459 (76.4)	ns	230 (76.2)	229 (76.6)	ns
Kidney disease	44 (5.5)	11 (5.6)	33 (5.5)	ns	20 (6.6)	13 (4.3)	ns
Cerebral haemorrhage	8 (1.0)	1 (0.5)	7 (1.2)	ns	3 (1.0)	4 (1.3)	ns
GI-bleeding	10 (1.3)	3 (1.5)	7 (1.2)	0.022	7 (2.3)	0 (0.0)	0.015
Anaemia	54 (6.8)	17 (8.7)	37 (6.2)	ns	19 (6.3)	18 (6.0)	ns
Atrial fibrillation	582 (73.1)	141 (72.3)	441 (73.4)	ns	207 (68.5)	234 (78.3)	0.007
CABG	140 (17.6)	43 (22.1)	97 (16.1)	0.032	45 (14.9)	52 (17.4)	ns
Previous PCI	197 (24.7)	46 (23.6)	151 (25.1)	ns	70 (23.2)	81 (27.1)	ns
**PCI-related information**							
Indication—ACS	536 (67.3)	125 (64.1)	411 (68.4)	ns	216 (71.5)	195 (65.2)	ns
Indication—angina pectoris	162 (20.4)	42 (21.5)	120 (20.0)	ns	48 (15.9)	72 (24.1)	0.012
Other indication	98 (12.3)	28 (14.4)	70 (11.6)	ns	38 (12.6)	32 (10.7)	ns
Elevated troponin	240 (30.2)	54 (27.7)	186 (30.9)	ns	95 (31.5)	91 (30.4)	ns
ST depression	140 (17.6)	28 (14.4)	112 (18.6)	ns	55 (18.2)	57 (19.1)	ns
Femoral artery access	294 (36.9)	89 (45.6)	205 (34.1)	0.004	106 (35.1)	99 (33.1)	ns
PCI with Stent	693 (87.1)	133 (68.2)	560 (93.2)	<0.001	286 (94.7)	274 (91.6)	ns
PCI with balloon✖	56 (7.0)	24 (12.3)	32 (5.3)	0.004	10 (3.3)	22 (7.4)	0.03
PCI other **	47 (5.9)	38 (19.5)	9 (1.5)	<0.001	6 (2.0)	3 (1.0)	ns
**Warfarin indication**							
Atrial fibrillation	564 (70.9)	130 (66.7)	434 (72.2)	ns	209 (69.2)	225 (75.3)	ns
Heart valve prosthesis	57 (7.2)	16 (8.2)	41 (6.8)	ns	23 (7.6)	18 (6.0)	ns
Venous thromboembolism	66 (8.3)	15 (7.7)	51 (8.5)	ns	25 (8.3)	26 (8.7)	ns
Other indication✉	233 (29.3)	50 (25.6)	183 (30.4)	ns	75 (24.8)	58 (19.4)	ns

Data is presented with number (percent), mean ± SD and median (IQR).

IQR = inter quartile range

*100 patients with warfarin and clopidogrel, 55 with warfarin and aspirin and 40 patients with warfarin alone

**Other = other therapy, atherectomy, brachytherapy, laser, wire only, rotablator

✖ POBA (plain old balloon angioplasty), drug eluting balloon and cutting balloon.

#Coronary- or peripheral vascular disease.

★ Creatinine clearance was calculated with Cockcroft-Gault. Men: (1.23 * (140—age) * weight) / S- Creatinine. Women: (1.04 * (140—age) * weight) / S- Creatinine. (Clinical Pharmacokinetics, Concepts and Applications, 3rd edition 1995, M Rowland)

✉Other indication to warfarin. E.g. myocardial infarction, cardiomyopathy and left ventricular thrombus.

**Table 2:** Cox regression analysis estimating univariate and multivariate HR for bleeding as outcome in 432 triple-treated patients with all included variables available.

Variables	Univariate HR (95% CI)	Multivariate HR* (95% CI)
iTTR<70	2.82 (1.30–6.01)	2.86 (1.25–6.53)
**Background data**		
Age at PCI	1.04 (1.00-1-09)	0.99 (0.89–1.10)
Male gender	0.38 (0.19–0.77)	0.87 (0.21–3.45)
History of smoking	0.62 (0.31–1.25)	0.66 (0.30–1.45)
Creatinine clearance	0.99 (0.98–1.00)	0.99 (0.97–1.01)
BMI	1.04 (0.97–1.11)	1.11 (1.01–1.22)
CHA_2_DS_2_-VASc	1.42 (1.17–1.72)	1.85 (0.57–6.03)
**Medical history**		
Heart failure	2.19 (1.08–4.43)	1.09 (0.25–4.68)
Hypertension	1.58 (0.65–3.85)	0.47 (0.10–2.19)
Diabetes	1.41 (0.68–2.92)	0.50 (0.12–2.16)
Stroke/TIA	1.54 (0.74–3.2)	0.36 (0.03–4.61)
Vascular disease	1.93 (0.74–5.02)	0.99 (0.20–4.81)
GI-bleeding	7.13 (1.70–29.89)	2.92 (0.57–14.98)
Anaemia	4.44 (1.83–10.81)	3.17 (1.16–8.69)
Atrial fibrillation	1.08 (0.47–2.51)	1.03 (0.42–2.52)
CABG	1.31 (0.59–2.94)	1.02 (0.42–2.51)
Previous PCI	0.88 (0.41–1.90)	0.80 (0.35–1.84)

Univariate HR = Hazard Ratio for one variable

*Multvariate HR = Hazard Ratio including all varibles

### Endpoints

Bleeding complications for triple treated patients from the time of the intervention until 183 days afterwards were extracted from the NPR by ICD-10 codes. Bleeding was defined according to the standardized bleeding definitions for cardiovascular clinical trials as BARC type 2, serious bleedings resulting in in-hospital care [28]. The bleedings were subdivided into cerebral, gastrointestinal and other bleeding, mainly anemia. The ISTH definition of serious bleeding could not be used due to lack of information regarding Hb reduction of 20g/L or transfusion of at least two units of blood, of which data was not possible to obtain from the NPR [29]. ICD-10 codes used for defining background characteristics and bleeding events are presented in the appendix.

### Statistical analysis

Statistical analysis was performed using SPSS Statistics (Version 24 for Mac; SPSS Inc., IBM Corporation, NY, USA). Calculation of time and percentage of time with correct International Normalized Ratio (INR) and additional calculations of the results (bleeding / 100 treatment years with confidence intervals) were made with Microsoft Excel for Mac 2011 (version14.4.5.141.003). Calculation of iTTR was made according to the Rosendaal method [30]. When a bleeding complication occurred, the INR value for the date of the bleeding was not used to calculate iTTR. This was because the effect of warfarin might have been reversed on that day leading to a false low iTTR. Descriptive background data is presented in Table 1 where the categorical variables are reported in number and percentage of the distribution within each treatment group. Categorical variables were compared with Chi-2 test where two-sided p-values were calculated. Continuous variables with normal distribution are reported as mean and standard deviation and compared using analysis of variance (ANOVA). Variables without normal distribution are reported as median and interquartile range (IQR) and compared with Kruskal-Wallis test. The results were analysed with Cox regression analysis and Chi-2 test or Chi-2 linear by linear when appropriate. The quality measure of the warfarin treatment in the form of iTTR was divided into iTTR above and below 70%, which is what The National Board of Health and Welfare in Sweden recommend [31]. A split of iTTR into quartiles gave: Q1: 53.3%, Md: 69.5%, Q3: 82.6%. From those quartiles clinically more useful groups where created were iTTR group 1 had a therapeutic INR (INR 2–3) <50.0% of the time of the study period. Group 2 is therapeutic 50 to 69.99%, group 3: 70–84.99% and group 4 ≥85% of the time.

Only the first bleeding complication during the study period was included in the calculation of the total outcome "bleeding" (a merger of other bleeding, gastrointestinal and cerebral bleeding). If an individual received an additional gastrointestinal or cerebral bleeding during the study period it was also recorded but not included in the calculation of the total outcome “bleeding”(Table 3). The bleeding rate was defined as number of bleeding complications / 100 treatment years.

**Table 3:** Outcome regarding BARC type 2 bleeding for the 601 triple treated patients divided into clinical iTTR groups.

iTTR-group (n)	<50% n = 126	50–69.9% n = 176	70–84.9% n = 176	>85% n = 123	Total n = 601	P-value
Bleeding total	17 (13.5)	11 (6.3)	7 (4.0)	4 (3.3)	39 (6.5)	0.003
Anaemia	14 (11.1)	8 (4.5)	5 (2.8)	4 (3.3)	31 (5.2)	0.007
GI-bleeding	3 (2.4)	0 (0.0)	0 (0.0)	1 (0.8)	4 (0.7)	0.046
Cerebral haemorrhage	1 (0.8)	4 (2.3)	3 (1.7)	0 (0.0)	8 (1.3)	ns
Bleeding rate (95% CI)	44.9 (38.4–51.5)	15.1 (11.4–18.9)	9.5 (6.5–12.6)	8.0 (4.7–11.3)	16.7 (14.6–18.8)	

Reported in number and percentage distribution within each iTTR group, n (%). Bleeding rate is defined as bleedings per 100 treatment years.

P value <0.05 was considered significant.

## Results

The final cohort consisted of 796 PCI-patients with an adequate warfarin period. 75.5% of those belonged to the group with triple therapy for which the analysis of outcome was intended. Background data for the entire cohort based upon the antithrombotic therapy chosen is presented in Table 1.

**Fig 2:**
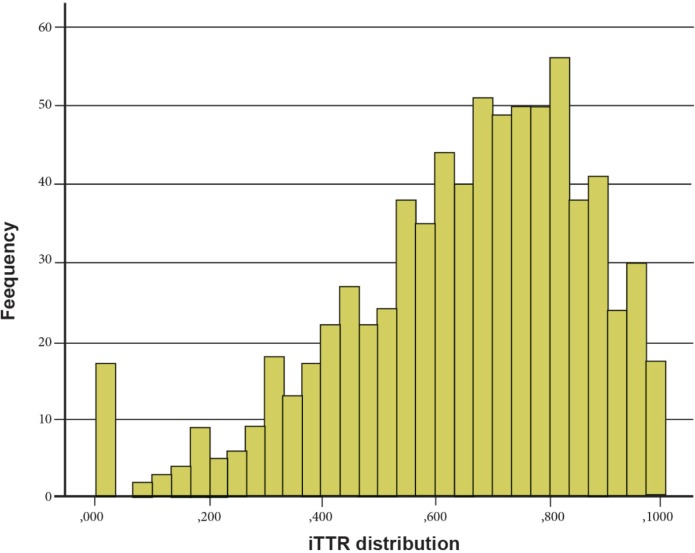
iTTR distribution.

The age distribution for the total cohort (796) ranged between 35 to 90 years with the distribution <65 years (22.7%) age 65 to 74.9 years (39.3%) and >75 years (37.9%). There was no difference in the antithrombotic therapy chosen according to age group (p = 0.307). The median iTTR in the cohort was 69.5% (53.4–82.6) (Fig 2). Women represented 24.1% of the total cohort and there was no difference in the choice of antithrombotic therapy according to gender (p = 0.942).

In the group with triple treatment, women constituted 24.0% and over half of the women were over 75 years of age. The age distribution for triple treated women was: <60 years: 16.7%. 65–74.9 years: 31.9% >75 years: 51.4%. The age distribution of triple-treated males was: <65 years: 26.3%, 65–74.9 years: 40.5%, >75 years: 33.3%. In all groups, atrial fibrillation was the most common indication for warfarin therapy (60–80%) and some patients had more than one indication for warfarin.

There was a trend towards higher bleeding frequency in the confirmed triple treated group of 601 patients (6.5%) vs. the remaining 195 patients (4.1%), however not significant (p = 0.22).

High age, female gender and a high CHA_2_DS_2_-VASc score as well as a medical history of heart failure and GI-bleeding increased the risk of bleeding in univariate Cox regression analysis, however none of these were significant in the multivariate analysis (Table 2). Univariate analysis for all triple-treated patients (n = 601) did not change the significance compared to the analysis of only those without missing data (n = 432) (data not shown). Low BMI slightly increased the risk of bleeding in multivariate analysis (HR 1.11, 95% CI 1.01–1.22). A medical history of anemia and an iTTR below 70% were strong independent risk factors for bleeding, HR 3.17 (1.16–8.69) and 2.86 (1.25–6.53) respectively.

The number of bleeding events in patients with triple therapy (n = 601) and iTTR >70% was 11 (3.7%) compared to 28 (9.3%) in patients with iTTR <70% (p = 0.005). Dividing iTTR into quartiles, better warfarin treatment quality corresponded to fewer bleeding events except cerebral hemorrhage (Table 3) The rate of bleeding complications in the group with the lowest warfarin treatment quality therapy, (iTTR <50%) was 44.9 / 100 treatment years whereas the rate for the best-treated group with iTTR >85% was 8.0 / 100 treatment years. The confidence interval for the group with iTTR <50% does not overlap with the other iTTR groups.

Men had a lower incidence of bleeding compared to women in the triple treated group, 4.8% versus 11.8% (p = 0.003). Women displayed a higher proportion of bleeding among elderly with the distribution: <65 years: 3.5%, 65–74.9 years: 6.1%, >75 years: 8.8%. (p = 0.038, Chi-2, linear by linear). As for the triple treated group (n = 601) the proportion of bleeding was gradually increasing for the whole cohort (n = 796) the lower the time within therapeutic INR (p<0.001).

## Discussion

Major bleeding complications are 1.5 to 2.5 times more common for triple therapy compared to a strategy with two platelet inhibitors, DAPT [32–34].

DAPT on top of oral anticoagulation therapy increase the risk of bleeding complications two- to threefold [35–37]. Aspirin as a single platelet inhibitor in addition to warfarin therapy also increases the risk of bleeding significantly [18]. The WOEST trial showed that by omitting aspirin, the risk of bleeding decreased [7]. Later studies such as PIONEER AF-PCI and RE-DUAL have shown that a combination of low dose NOAC and P2Y12 inhibitor also results in a lower risk of bleeding compared to triple therapy [38, 39]. If the individual time in therapeutic range (iTTR) is below 70% it has previously been shown that the risk for a major bleeding is increased in patients with atrial fibrillation, venous thromboembolism or mechanical heart valve prosthesis [18–21]. Our study shows that the patient’s iTTR also is important for the rate of bleeding complications during triple therapy after PCI. In this study iTTR<70% almost triples the risk of a BARC type 2 bleeding complication compared to good warfarin treatment quality with iTTR>70%. An American study with similar baseline characteristics and nearly the same number of patients on triple therapy as in our study, showed that 10.9% had a bleeding complication that involved rehospitalisation during a period of six months [6]. If no consideration is taken to iTTR the corresponding figure in this study was 6.5% for triple therapy. It has also been shown that DAPT after PCI even without warfarin therapy can cause 13.8% BARC 2 bleedings during a period of six months [40]. In this study 13.3% had a BARC 2 bleeding in the worst group with iTTR<50% whereas only 3.3% had a bleeding complication in the group with iTTR>85% during a period of six months (p = 0.003). Apart from a clinical history of anemia, iTTR is the most important, and still a modifiable predictor of bleeding risk. The rate of bleeding increases with lower proportion of time within therapeutic INR. This is also evident when taking the time until bleeding in consideration, calculating bleeding rates. The two groups with the best quality of their warfarin treatment (iTTR 70–84.9 and >85%) had a low bleeding rate, 9.6 and 8.0 respectively, while the worst group with iTTR <50% had a much higher bleeding rate of 44.9 per 100 treatment years (p = 0.003). Triple-treated women had a higher proportion of bleeding compared to men in our study (11.8% vs. 4.8%, p = 0.003). It is likely that the age distribution to some extent contributes to this outcome. Age was itself a risk factor for bleeding (p = 0.038, Chi-2, linear by linear) and we found that over half of the triple-treated women were 75 years or older compared to men where 33% were ≥75 years.

### Limitations

This is an unselected cohort in a “real life” observational study. As in all research based upon registry data this study has a risk of confounding. Comparisons between observation studies and randomized studies may be interpreted with caution.

As an effort to get a representative iTTR for the patients, we did not include the INR value the day of the complication since this could be falsely low after a possible warfarin reversal. We might thereby have missed a high INR value the day of the bleeding, which however only would have increased the bleeding incidence in the lower iTTR groups. Although the quality of data in Swedish registries maintains a high standard by international comparison, there is always a risk that data has been entered incorrectly or is missing. As an effort to reduce the risk of getting non-triple treated patients in the cohort we used a time interval for the drug-dispensing event in connection to the intervention (PCI procedure). We can not be certain that the patients actually took their medication. On the other hand, since the risk of stent thrombosis is very high without adequate platelet inhibition during the immediate period after stent implantation it seems more likely that they took their prescribed medication. Because of lack of data, this study did not look into whether there was a relationship between the outcome of this study and established risk scores predicting bleeding such as HAS-BLED, Mehran and the Crusade [41–43]. It is likely that some of the cases with anaemia are undiagnosed GI bleedings, accounting for the high bleeding risk in this group. The relative importance of iTTR<70% compared to background characteristics regarding risk of bleeding complication was performed although iTTR is a variable measured during the study period and not known at the date of the PCI.

## Conclusion

Triple therapy after PCI increases the rate of bleeding and this is particularly evident when the quality of warfarin therapy is low measured by iTTR. Low iTTR <70% and a history of previous anemia are strong independent risk factors almost tripling the risk for BARC type 2 bleeding. On the other hand, patients on triple therapy with high iTTR show a lower bleeding rate than previously reported in patients with oral anticoagulation and only one thrombocyte inhibitor. Since warfarin treatment quality is a modifiable risk factor, attention should be paid to iTTR in patients on triple therapy after PCI.

## Supporting information

S1 Appendix (DOCX)

S1 Rawdata (SAV)

S2 Rawdata (XLSX)

## Abbreviations

ACS: acute coronary syndrome
ADP: adenosine diphosphate
ANOVA: Analysis of Variance
AF: atrial fibrillation
BARC: Bleeding Academic Research Consortium
BMI: body mass index
CI: confidence interval
CABG: coronary artery bypass grafting
Chi-2: Chi-Square test
CHA_2_DS_2_-VASc: Congestive heart failure or left ventricular dysfunction, Hypertension, Age ≥75 [Doubled], Diabetes, Stroke [Doubled]–Vascular disease, Age 65–74 and Sex category [Female]
CRUSADE: Can Rapid risk stratification of Unstable angina patients Suppress ADverse outcomes with Early implementation of the ACC/AHA Guidelines
DAPT: dual antiplatelet therapy
FFR: fractional flow reserve
GI: gastrointestinal
HAS-Bled: Hypertension, Abnormal renal/liver function, Stroke, Bleeding history or predisposition, Labile INR, Elderly, Drugs/alcohol
ICD: International Statistical Classifications of Diseases
INR: international normalized ratio
ISTH: International Society on Thrombosis and Haemostasis
ITTR: individual time in therapeutic range
IVUS: intravascular ultrasound
IQR: inter quartile range
Md: median
NOAC: non-vitamin K antagonist oral anticoagulant
NPR: The Swedish National Patient Registry
PCI: percutaneous coronary intervention
POBA: plain old balloon angioplasty
SCAAR: Swedish Coronary Angiography and Angioplasty Registry
SD: standard deviation
SWEDEHEART: Swedish Web System for Enhancement and Development of Evidence-Based Care in Heart Disease Evaluated According To Recommended Therapies
TIA: transient ischemic attack
WOEST: What is the Optimal antiplatElet and anticoagulant therapy in patients with oral anticoagulation and coronary StenTing
