# Supplementary material for: Triple therapy after PCI – Warfarin treatment quality and bleeding risk
Source: PLoS One. 2018 Dec 18;13(12):e0209187. doi: 10.1371/journal.pone.0209187 (PMC6298652; doi:10.1371/journal.pone.0209187)
Supplement: S1 Appendix — (DOCX) [file pone.0209187.s001.docx]

**APPENDIX - ICD-10-codes**

| **Background data:** |  |
| --- | --- |
| Diabetes | E10-14 |
| Hypertension | I10–15 |
| Stroke/TIA | I63-64, G45 (not G45.4), I69 |
| Heart failure | I50, I11, I130, I132 |
| Kidney disease | I120, I131-132, N17-19, DR016, DR024, KAS00, KAS10, KAS20 |
| Stroke/TE/TIA | I63-64, I74, G45 |
| Venous thromboembolism | I26, I636, I676, I80–82 |
| Myocardial infarction Peripheral vascular disease  Atrial fibrillation | I21–22, I24, I25.2  I70-73  I48 |
| **Background and Outcome data**  Bleeding: |  |
| Cerebral haemorrhage | I60-I62, S064–066 |
| GI-bleeding | I850, I983, K250, K252, K254, K256, K260, K262, K264, K266, K270, K272, K274, K276, K280, K282, K284, K286, K625, K920–922 |
| Other | D500, D508-509, D629, H365, H922, N02, N938–939, R04, R310 |
